# Current Understanding of the Neurobiology of Agitation

**DOI:** 10.5811/westjem.2020.4.45779

**Published:** 2020-07-02

**Authors:** Christopher W.T. Miller, Vedrana Hodzic, Eric Weintraub

**Affiliations:** University of Maryland School of Medicine, Department of Psychiatry, Baltimore, Maryland

## Abstract

**Introduction:**

Managing agitation in the clinical setting is a challenge that many practitioners face regularly. Our evolving understanding of the etiological factors involved in aggressive acts has better informed our interventions through pharmacologic and behavioral strategies. This paper reviews the literature on the neurobiological underpinnings of aggressive behaviors, linking psychopathology with proposed mechanisms of action of psychiatric medications shown to be effective in mitigating agitation.

**Methods:**

We performed a review of the extant literature using PubMed as a primary database. Investigation focused on neurobiology of agitation and its relation to the current evidence base for particular interventions.

**Results:**

There are well-established pathways that can lead to increased autonomic response and the potential for violence. Psychopathology and substance-induced perceptual distortions may lead to magnification and overestimation of environmental threat, heightening the potential for aggression. Additional challenges have arisen with the advent of several novel drugs of abuse, many of which lead to atypical clinical presentations and which can elude standard drug screens. Our interventions still lean on the evidence base found in Project BETA (Best Practices in Evaluation and Treatment of Agitation). Although not a new drug and not included in the Project BETA guidelines, ketamine and its use are also discussed, given its unique pharmacology and potential benefits when other protocoled interventions have failed.

**Conclusion:**

Aggression can occur due to manifold reasons in the clinical setting. Having an informed understanding of the possible determinants of agitation can help with more tailored responses to individual patients, limiting the unnecessary use of medications or of interventions that could be deemed forceful.

## INTRODUCTION

Managing agitation in acute clinical settings is a challenge for many practitioners. There have been exciting advances in the neurosciences over the past few decades, allowing for some correlation between what is observed clinically and underlying neuroendocrine alterations leading to aggressive behaviors. While our pharmacologic tools have not necessarily progressed at the same pace, there is a greater appreciation of how particular interventions work on a neurobiological level. Importantly, there is also an awareness of the limitations to some of our forms of treating aggression. One emerging challenge is the use of novel substances of abuse, many of which elude traditional drug screening (e.g., synthetic cannabinoids).^1^,^2^ Thus, practitioners may see how patients are presenting, yet do not have the tools at their disposal to make a more precise diagnosis. We review the extant literature on the neurobiological underpinnings of aggressive behaviors, linking psychopathology with proposed mechanisms of action of psychiatric medications shown to be effective in mitigating agitation.

## METHODS

We performed a literature search using the PubMed electronic database looking for English-language research articles addressing the neurobiology of agitation and its management. No start date limitations regarding the date of publication were employed. The search was limited to articles published by October 29, 2019, the last date the search was conducted. In order to discuss the neurobiological underpinnings of agitation and how this correlates with current, evidence-based, treatment options, we selected the following search terms as title/abstract words, independent terms, text words, or medical subject headings (MeSH) terms and subsequently combined them with the Boolean term “and” 1) *neuroscience* or *neurobiology*; 2) *medications* or *psychiatric medications* or *psychopharmacology*; 3) *emergency medicine* or *emergency psychiatry*; and 4) *agitation* or *aggression* or *violence*. Additionally, we conducted hand searches of reference lists of selected articles for this review to identify other relevant articles. The search strategy was performed by one of the authors (CM).

Out of 5,641 articles yielded by the search, further review of titles and abstracts for relevance to the topic of this paper reduced the number to 480. To maintain focus on the neurobiology of agitation and the psychopharmacologic interventions currently in clinical use, we excluded studies evaluating non-pharmacologic interventions – other than to recognize their importance and evidence base. Following the exclusion of such articles and further review of the studies assessing key results and limitations, 55 articles were selected for final inclusion.

## RESULTS AND DISCUSSION

### Neuroscientific Underpinnings of Agitation

There are well-established pathways that can lead to increased threat perception, autonomic response, and aggression. The amygdala, a component of the limbic system, is sensitive to signs of threat and reciprocally innervates areas involved in salience-driven responses (e.g., locus coeruleus [LC], bed nucleus of the stria terminalis [BNST], anterior insula, periaqueductal gray [Pag], and hypothalamus).^3^,^4^ These connections regulate stress hormone release. Efficient coupling of higher cortical areas (e.g., medial prefrontal cortex [mPFC], orbitofrontal cortex [OFC] and anterior cingulate cortex [ACC]) with limbic regions modulates top-down inhibitory control.^5^ This allows for risk-reward considerations and calibration of behaviors to social cues prior to engaging in action. This permits responses that are not excessively driven by immediate salience and affective tone.

However, psychopathology and substance-induced perceptual distortions may lead to erroneous interpretation of environmental stimuli and overestimation of threat, heightening the potential for aggression. In such instances there is an excessive bottom-up activation, with insufficient behavioral control from higher cortical regions. Some psychiatric disorders (e.g., borderline and antisocial personality disorders) are notable for hypoactivity in cortical areas, leading to a default of affectively-driven behavioral reactions, with little recourse for deploying more adaptive strategies.^6^,^7^

Overestimation of environmental threat, through activation of the hypothalamic-pituitary-adrenal axis, causes excessive release of catecholamines (e.g., norepinephrine and dopamine), glutamate, and acetylcholine. Aggressive states are also marked by diminished levels of serotonin and gamma-amino-butyric acid (GABA), both of which are involved in the top-down control of limbic activation; indeed, these latter two neurotransmitter systems develop in tandem early in life, informing one’s ability to modulate dysphoric reactions. This is conceptually important, as fear activation pathways and the circuitry involved with aggressive responses demonstrate considerable overlap.^8^ An individual experiencing a behavioral emergency may experience the environment as unsafe and deploy strategies deemed necessary to ensure survival. The amygdala expresses adrenoreceptors and dopamine D2 receptors, and there are direct projections onto amygdalae nuclei from both the ventral tegmental area and LC, brainstem areas responsible for synthesis and release of dopamine and norepinephrine, respectively. Increase in these catecholamines (as seen, for instance, in acute psychosis, mania, and stimulant intoxication) can increase amygdala excitation, exacerbating conditioned fear responses and paranoia, which may reach delusional proportions.^9^,^10^

In addition to these subcortical effects, excess in norepinephrine release may bias cortical activation toward more posterior and inferior areas. The PFC has reciprocal connections with the LC, modulating tonic activity in the latter and thus regulating norepinephrine release.^11^ Optimal levels of norepinephrine are important for appropriate PFC activity, including working memory and executive functioning. Of the three families of noradrenergic receptors (α1, α2, and β), norepinephrine has the highest affinity for α2,^12^ with α2A being the most abundant subtype located in the PFC.^13^,^14^ Thus, in low-stress situations α2 receptors are engaged preferentially, allowing for access to PFC functioning, including control of limbic activity. As stress levels rise and more norepinephrine is released, α1 and β receptors are engaged, and an individual’s ability to think and consider different behavioral options may be diminished. Interventions aimed at decreasing autonomic arousal and the consequent behavioral overtones that might ensue can include anti-adrenergic drugs such as propranolol (a non-receptor-specific beta-blocker); the latter has been shown to be effective in conditions such as intermittent explosive disorder and aggressive behaviors associated with traumatic brain injuries, in which agitation may be out of proportion to the inciting stimulus.^15^–^17^

Produced in the dorsal and median raphe nuclei, serotonin is a predominantly inhibitory neurotransmitter, shown to be involved with controlling aggressive behaviors directed at self and others. Low levels of the serotonin metabolite 5-hydroxyindoleacetic acid have been demonstrated in the cerebrospinal fluid of individuals with aggressive personality traits and in those who have attempted suicide by violent means.^18^,^19^

While it is beyond the scope of this review to cover all substances of abuse in detail, it should be noted that many recreationally used drugs have complex mechanisms of action that accentuate autonomic drive, threat perception, and limbic-based behavioral responses. Stimulants such as cocaine and amphetamines work predominantly as norepinephrine-dopamine reuptake inhibitors. Some of the phenylethylamines, such as methylenedioxymethamphetamine and methamphetamine, also have serotonergic properties, with the potential for long-term neurotoxic effects on the serotonin pathway, resulting in impulsive and aggressive behaviors, due to insufficient top-down modulation. For instance, methamphetamine use has been associated with decreased serotonin transporter density in the OFC and ACC, a finding correlated with increased levels of aggression.^20^ Some ergoline (e.g., lysergic acid diethylamide [LSD]) and tryptamine (e.g., dimethyltryptamine) hallucinogens may act as mixed 5-HT1A/5-HT2A agonists, leading to imbalance in excitatory/inhibitory glutamatergic transmission, with potential for sensory distortions and threat magnification. Also, LSD has been shown to possess intrinsic activity at striatal D2 receptors, which may contribute to euphoria, depersonalization, and psychotomimetic effects.^21^

A growing concern is with synthetic blends of cannabinoids, many of which possess stronger activity at cannabinoid receptor 1 (CB1) compared to tetrahydrocannabinol. CB1 is G-protein linked and primarily pre-synaptic; it is involved with regulating neuronal release of neurotransmitters such as glutamate and catecholamines; this helps control neuronal excitability. The use of exogenous cannabinoids may disrupt this process, resulting in excessive glutamatergic and dopaminergic tone, leading to anxiety, paranoia, and psychotic symptoms in susceptible individuals.^22^ The potential for adverse effects is furthered with the synthetic blends, which in many instances *lack cannabidiol*, a component with antipsychotic and antiepileptic properties.^23^

### Management of Agitation – Pharmacological Options

In 2012 the American Association of Emergency Psychiatry put forth evidence-based guidelines for treatment of agitation, termed Project BETA (Best Practices in the Evaluation and Treatment of Agitation).^24^ While an extensive review of these guidelines is beyond the scope of this paper, we have attempted to discuss the utility of particular interventions in light of the neurobiological considerations mentioned previously. Importantly, agitation may be multifactorial. Medical causes (e.g., hypoglycemia, hypoxia, ictal phenomena) should always be considered, as treating the underlying etiology is the intervention of choice in such situations.^25^

When treating agitation of unclear etiology, the first-line treatment is benzodiazepines,^24^ many of which have considerable advantages in terms of route of administration and predictability of onset. Lorazepam has the additional benefit of not undergoing stage I hepatic oxidation, making it an attractive option when liver function may be relevant but cannot be gauged. Acting as GABA-A receptor agonists, benzodiazepines can aid with top-down cortical-limbic inhibitory control. GABA is the main inhibitory neurotransmitter in the central nervous system (CNS), influencing 60–70% of all synapses.^26^ Inhibitory coupling of areas of the PFC with the amygdala is mediated by GABAergic interneurons,^27^ which may be compromised in agitated states. In states of dysphoria, this inhibitory circuitry may be entirely bypassed in favor of a more direct activation of the central amygdala, leading to less flexible behavioral and affective responses.^28^ Benzodiazepines are also the treatment of choice in clinical scenarios in which there is a relative deficiency of GABAergic tone, leading to autonomic and behavioral symptoms (e.g., withdrawal from alcohol or from chronic benzodiazepine use). In cases where known GABA-A agonists (e.g., alcohol) may be *causing* behavioral activation, providers should *refrain* from using benzodiazepines, as exposure to additional GABA-A agonism may promote further disinhibition.^29^ In line with this, the treatment of choice for agitation due to alcohol intoxication is haloperidol, per Project BETA.^24^

Acute management of aggression in the context of psychosis aims to decrease stimulation that could be perceived as menacing, as well as to provide medications that are sedating and anxiolytic. Antipsychotic drugs, with few exceptions, display D2-blocking properties and have variable adrenoreceptor binding properties, modulating CNS adrenergic neurotransmission. As antipsychotics have been shown to bind to the amygdala,^30^ these mechanisms of action may help mitigate catecholaminergic drive and threat perception. Project BETA recommends atypical antipsychotics (e.g., risperidone or olanzapine) as first line for psychosis-driven agitation, with or without addition of a benzodiazepine. Second-line treatment would consist of a typical agent (e.g., haloperidol) in combination with a benzodiazepine.^24^ The preference for atypical antipsychotics may derive from their receptor-binding profile, providing clinical benefit with less propensity for extrapyramidal symptoms (EPS). Indeed, using high-potency, typical antipsychotics usually warrants concomitant use of an anticholinergic agent to prevent EPS,^31^ although this has been debated in the literature.^32^

**Figure 1:**
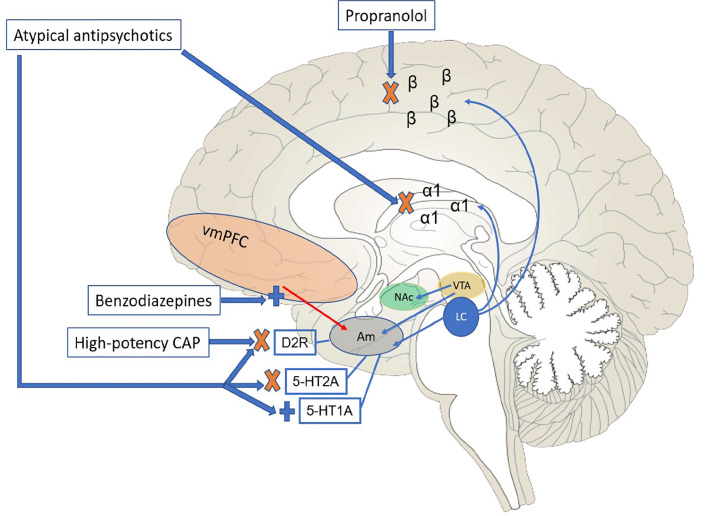
Schematic depiction of medial surface of cortex, subcortical areas, and brainstem, demonstrating key neural circuitry linked with the mechanisms of action of drugs used for the treatment of acute agitation. In states of heightened catecholaminergic tone, such as stimulant intoxication, acute psychosis, or mania, there may be excessive dopamine availability, binding amygdala D2 receptors and increasing conditioned fear responses. In addition, noradrenergic input from the locus coeruleus may also be elevated, contributing to autonomic arousal and feelings of paranoia. As levels increase, binding of norepinephrine will be shifted from the prefrontal cortex (PFC) to posterior cortical and subcortical regions (indicated by β and α1 receptors – schematically depicted for didactic simplicity), decreasing the individual’s ability to cognitively negotiate the situation at hand, particularly as PFC-amygdala coupling is diminished. Medications used for agitation can mitigate the effects of this neurotransmitter and circuitry make-up through the following mechanisms: 1) benzodiazepines, through GABA-A agonism, increase the PFC inhibitory control over the amygdala; 2) beta-blockers (e.g., propranolol, an agent with considerable lipophilicity), in addition to their peripheral effect on autonomic arousal, can decrease norepinephrine binding to posterior adrenoreceptors, thus allowing for greater PFC binding; (3) conventional, or typical antipsychotics, particularly the high-potency agents (e.g., haloperidol), work primarily through D2 receptor blockade – this occurs within the striatum, but also in the amygdala, decreasing threat perception; 4) atypical antipsychotics have a complex mechanism of action – a) D2 blockade occurs, though therapeutic occupancy is less than required with typical agents, b) several act as α1 receptor antagonists, decreasing subcortical adrenoreceptor binding, c) through subcortical serotonergic modulation, anxiolysis is promoted – several atypicals (e.g., clozapine, ziprasidone, lurasidone, quetiapine, and aripiprazole) agonize the Gi-linked (inhibitory) 5-HT1A receptor and all atypicals antagonize the Gq-linked (excitatory) 5-HT2A receptor, thus diminishing amygdala activation. *Am*, amygdala; *CAP*, conventional (first-generation) antipsychotic; *D2R*, dopamine 2 receptor; *LC*, locus coeruleus, *NAc*, nucleus accumbens; *vmPFC*, ventromedial prefrontal cortex; *VTA*, ventral tegmental area.

In addition to generally showing higher antagonistic affinity for histamine-1 receptors as compared to typical agents,^33^ thus providing more sedative effects, atypical antipsychotics antagonize 5-HT2A. This mechanism can decrease excitatory glutamatergic tone as well as increase local release of dopamine in the nigrostriatal pathway, thus providing some protection against EPS. These ancillary mechanisms are important, as they may factor more into immediate behavioral control than D2-blocking properties, which require longer-term use to achieve appropriate receptor occupancy and full clinical effect. It should be noted that this wider receptor profile is also shared to some extent by lower-potency typical antipsychotics (e.g., chlorpromazine), which have clinical use in management of agitation. However, their side-effect profiles can limit more regular use; for instance, chlorpromazine has been associated with significant orthostatic hypotension, particularly with parenteral formulations. Figure 1 schematically depicts the neural pathways and relevant medication effects discussed thus far.

Of note, Project BETA guidelines for agitation in psychosis also apply for individuals who have a diagnosis of bipolar disorder and are presenting with acute mania. As drugs such as lithium and antiepileptics may take up to two weeks to achieve a steady state, more immediate behavioral control with antipsychotics (with or without benzodiazepines) may be necessary. One important caveat applies with regard to mania. Despite being approved by the US Food and Drug Administration (FDA) for control of mania, as well as being listed in Project BETA guidelines as a third-line agent for agitation in such scenarios, ziprasidone (an atypical antipsychotic) may lead to enhanced adrenergic output and serotonergic neurotransmission, working in effect as a serotonin-norepinephrine reuptake inhibitor, which could ostensibly worsen a patient’s symptoms if such activation is not offset by the sedative properties of the drug.^34^

With regard to delirium, a wide differential diagnosis of possible medical conditions should be kept in mind. While environmental interventions are imperative to mitigate agitation and worsening of confusion in the patient (e.g., controlling sensory stimuli, early mobility, nutrition, and providing frequent reorientation), certain pharmacologic principles should be heeded. Delirium is, in effect, a hyperdopaminergic and anticholinergic state. Despite this neurobiological substrate, there is *not* compelling evidence for use of antipsychotics for prophylactic prevention of delirium,^35^ and recent evidence has questioned whether use of antipsychotics actually leads to improved outcomes in patients with *established* delirium.^36^ As such, if antipsychotics are necessary, their use should be short-term and limited to situations in which delirium is accompanied by behavioral dyscontrol.^37^ When selecting an antipsychotic, the suggested approach is the use of typical antipsychotics, ideally those with high D2-blocking potency and low intrinsic anticholinergic properties (e.g., haloperidol).^24^ Given the pathology-driven anticholinergic tone in this condition, there is relative protection against EPS, allowing for high doses of antipsychotics to be given.^38^ At such doses, it has been suggested that haloperidol, in addition to its D2-blocking properties, may also have antioxidant properties due to interaction with opioid receptors. Benzodiazepines should also be avoided (except for management of substance withdrawal),^35^,^37^ given the potential for worsening of the clinical picture.

Managing agitation in patients with dementia should be largely non-pharmacological, when possible, as there is an evidence base supporting a number of such interventions.^39^,^40^ In Alzheimer dementia, in addition to the decline noted in cholinergic neurotransmission, the accumulation of amyloid plaques and neurofibrillary tangles may result in glutamatergic release, with potential for excitotoxicity. Available pharmacologic options include atypical antipsychotics (e.g., risperidone and olanzapine), antiepileptics (e.g., carbamazepine, gabapentin), serotonergic agents, and even less conventional options such as dextromethorphan/quinidine combination, the latter possessing antiglutamatergic properties.^41^–^44^ However, there is limited efficacy in use of pharmacologic agents, and the side-effect profile needs to be carefully weighed against potential benefits.^40^ For instance, atypical antipsychotics can lead to excess sedation, metabolic side effects, and EPS. In addition, a boxed warning from the FDA alerts prescribers to the increased risk of death associated with use of atypical antipsychotics in elderly patients with dementia-related psychotic symptoms.

Finally, we briefly discuss treatments with unique routes of administration or mechanisms of action. Alternative forms of administering medications may prove necessary when patient compliance or tolerance for oral medications is limited. An inhaled formulation of loxapine, a mid-potency typical antipsychotic, was approved in 2012 by the FDA for control of agitation associated with schizophrenia and bipolar disorder. While showing effectiveness in management of agitation,^45^ providing this medication effectively requires a considerable degree of patient cooperation, which may not always be feasible. In addition, the recommended dosing is limited to a one-time 10-milligram (mg) administration per 24-hour period, with patients requiring monitoring for bronchospasm for one hour after use.

**Figure 2:**
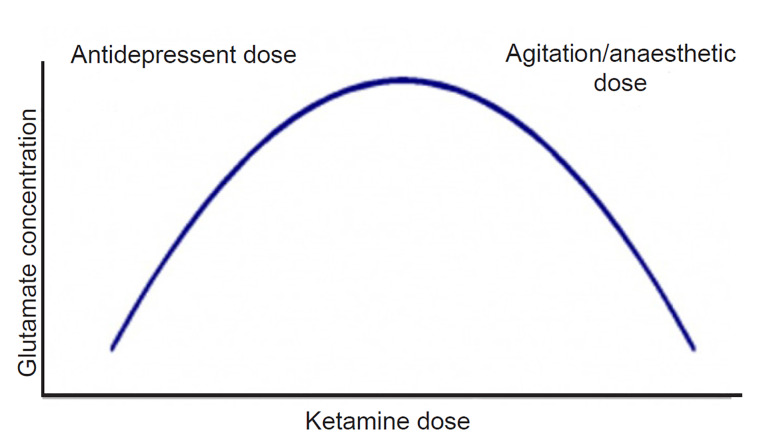
Depiction of dose-dependent inverted “U-shaped” curve associated with ketamine. Doses of ketamine used in antidepressant trials (0.5mg/kg) are associated with heightened down-stream glutamatergic neurotransmission, enhancing AMPA receptor activity. As doses increase toward those used in anesthesia and agitation (2–4mg/kg), there is a depression of glutamatergic tone, as well as an accretion of additional pharmacodynamic effects, including binding of opioid receptors. Thus, higher doses are typically required for behavioral control.

The use of ketamine, a non-competitive antagonist at glutamatergic N-methyl-D-aspartate (NMDA) receptors, has gained considerable interest in psychiatric practice. Ketamine has a complex mechanism of action, showing different pharmacodynamic receptor profiles and clinical effects according to the dose administered.^46^ While trials assessing antisuicidal and antidepressant properties typically employed doses of 0.5 mg per kilogram (kg),^47^ ketamine can be used in either intramuscular or intravenous formulations at doses of 2–4 mg/kg to control agitation. Pre-clinical and human studies have suggested that ketamine pharmacology follows an inverted “U-shaped” curve (Figure 2); in effect, at lower doses (used in depressive disorders), it can lead to a “glutamatergic burst,” given augmentation of α-amino-3-hydroxy-5-methyl-4-isoxazolepropionic acid (AMPA) receptor transmission.^48^ At progressively higher doses, there is a decrease in glutamatergic tone, as well as binding to mu and sigma-opioid receptors.^46^ This accounts for the higher dosage requirement in agitation as compared to those used in depression and suicidality. It has the important advantages of *not* a) increasing intracranial pressure (ICP) (indeed, it may actually *decrease* ICP in some cases);^49^,^50^ b) causing respiratory depression;^51^ or c) leading to clinically significant hemodynamic changes.^52^ However, its use can be limited by its dissociative effects, which become more pronounced at the higher doses that may be required depending on the degree of agitation. It has a rapid onset of action, and may be an attractive option for cases of severe agitation, especially when the etiology is unknown.

One study assessed the need for re-dosing of medications for agitation across a spectrum of diagnostic categories.^53^ Control of agitation was not always optimal when ketamine was the first drug used (although dosage varied considerably, from 40 mg – 400 mg); however, when used in refractory cases – poorly responsive to benzodiazepines and/or antipsychotics – ketamine had remarkable efficacy, with no cases requiring additional drug administration in the following three hours. Of note, the efficacy and safety of ketamine in geriatric patients and in individuals with neuropsychiatric disorders are still being investigated and need further study, although some small studies suggest lack of significant adverse effects in these populations.^54^,^55^ Finally, because doses of ketamine used to control behavior are more likely to lead to considerable sedation, with arguably less leeway to titrate to a minimal effective dose to reach a more measured state of calmness, it could be posited that this drug should not be considered as a first-line strategy, but rather reserved for refractory cases in which a rescue or second-line medication is needed.

## LIMITATIONS

While this paper provides an overview of the main neuroscientific aspects underlying agitation in clinical settings, it is difficult to account for atypical presentations, and there are instances informed by medical co-morbidity and pharmacologic side effects that were not covered in this paper. Also, despite the exciting developments in the neurosciences, they are still somewhat in their nascency, and any attempt to draw neurobiological parallels with clinical presentations will necessarily be limited by gaps and contradictions in the extant literature. While there is an evidence base that many of our interventions for agitation are effective, hopefully future research will allow for more tailored management, optimizing behavioral control while minimizing side effects.

## CONCLUSION

Aggression can present for manifold reasons in the clinical setting. Having a more informed understanding of the possible determinants of agitation can help with targeted treatment strategies, limiting the unnecessary use of medications or of interventions that could be deemed forceful. Decreasing catecholaminergic drive and/or augmenting GABAergic tone are particularly relevant considerations in management of agitation and are mechanistically germane to the treatment options posited by Project BETA. Although not discussed in this paper, it should be reiterated that non-pharmacological interventions are still essential considerations, in particular as the recovery model has been increasingly promoted to assist patients to feel a greater sense of control and partnership in the management of their care, even when they engage in violent acts in clinical settings.
